# Cross-clade neutralization of HIV-1 by a monoclonal antibody obtained by immunization with liposomes containing lipid A and a synthetic MPER peptide

**DOI:** 10.1186/1742-4690-9-S2-P4

**Published:** 2012-09-13

**Authors:** GR Matyas, O Jobe, L Wieczorek, KK Peachman, Z Beck, VR Polonis, M Rao, CR Alving

**Affiliations:** 1 Walter Reed Army Institute of Research, Silver Spring, MD, USA; 2 MHRP, WRAIR and Henry Jackson Foundation, Silver Spring, MD, USA

## Background

Development of vaccine formulations that induce cross clade neutralizing antibodies to the membrane proximal external region (MPER) of gp41 similar to 4E10 and 2F5 have been extensively investigated. We previously demonstrated that immunization with liposomes containing MPER and lipid A as an adjuvant could induce antibodies to the MPER and IgM monoclonal antibodies obtained from the mice bound to both lipids and MPER and neutralized HIV-1 (Matyas et al., 2009 AIDS 23, 2069-77). We now report a new IgG monoclonal antibody WR324 obtained from mice immunized with a similar vaccine formulation that binds to MPER and can neutralize multiple clades of HIV-1.

## Methods

Mice were immunized with liposomes containing phosphatidyl inositol-4-phosphate and MPER with lipid A as an adjuvant. The peptide, LELDKWASLWNWFDITNWLWYIK (aa 661-684) was derived from gp41 (HXB2 stain). Spleen cells were fused with SP2/O cells and cloned. WR324 was purified and the binding specificities were assessed by ELISA. HIV-1 neutralization was assessed in PBMC using replication competent, Renila reniformis luciferase (LucR)-expressing HIV-1 reporter viruses (LucR-IMC) and in a human monocyte derived macrophage (MDM) assay with purified viruses.

## Results

WR324 is an IgG2b with a kappa light chain and it bound to the MPER peptide, but not to lipids or recombinant gp41. WR324 neutralized clade B, BaL and SF162, clade CRF01_AE, CM235, and clade C, GS 014 IMC viruses in the PBMC assay using two different donors. It also neutralized primary viruses, US-1, BaL, and clade CRF01_AE, M066 in the MDM assay using 3 different donors. WR324 did not bind to the surface of MDM cells, but binding to HIV-1 was observed by electron microscopy with immuno-gold labeling.

## Conclusion

Immunization with liposomes containing MPER and lipid A as an adjuvant induced an IgG monoclonal antibody with specificity to the MPER that neutralized multiple clades of HIV-1 in two different assay systems.

